# Farrerol Ameliorates TNBS-Induced Colonic Inflammation by Inhibiting ERK1/2, JNK1/2, and NF-κB Signaling Pathway

**DOI:** 10.3390/ijms19072037

**Published:** 2018-07-13

**Authors:** Xin Ran, Yuhang Li, Guangxin Chen, Shoupeng Fu, Dewei He, Bingxu Huang, Libin Wei, Yuanqing Lin, Yingcheng Guo, Guiqiu Hu

**Affiliations:** 1 College of Animal Science and Veterinary Medicine, Jilin University, Changchun 130062, China; ranxin9914@mails.jlu.edu.cn (X.R.); yhli9915@mails.jlu.edu.cn (Y.L.); gxchen51143@163.com (G.C.); fushoupeng@jlu.edu.cn (S.F.); m13144303829@163.com (D.H.); huanbingxu123@163.com (B.H.); 2 Jiangsu Province Science and Technology Bureau, Taicang 215400, China; shoupengfu@163.com; 3 Animal Disease Prevention and Control Center of Qinghai Province, Xining 810001, China; lin_yq000@163.com; 4 Jilin Fengman Area Animal Epidemic Prevention and Control Center, Jilin 132013, China; hongqixumu@163.com

**Keywords:** Farrerol, IBD, colitis, MAPK, NF-κB

## Abstract

Farrerol, a type of 2, 3-dihydro-flavonoid, is obtained from Rhododendron. Previous studies have shown that Farrerol performs multiple biological activities, such as anti-inflammatory, antibacterial, and antioxidant activity. In this study, we aim to investigate the effect of Farrerol on colonic inflammation and explore its potential mechanisms. We found that the effect of Farrerol was evaluated via the 2,4,6-trinitrobenzene sulfonic acid (TNBS)-induced colitis model in mice and found that Farrerol has a protective effect on TNBS-induced colitis. Farrerol administration significantly improved the weight change, clinical scores, colon length, and intestinal epithelium barrier damage and markedly decreased the inflammatory cytokines production in TNBS-induced mice. The protective effect of Farrerol was also observed in LPS-induced RAW264.7 cells. We found that Farrerol observably reduced the production of inflammatory mediators including IL-1β, IL-6, TNF-α, COX-2, and iNOS in LPS-induced RAW264.7 cells via suppressing AKT, ERK1/2, JNK1/2, and NF-κB p65 phosphorylation. In conclusion, the study found that Farrerol has a beneficial effect on TNBS-induced colitis and might be a natural therapeutic agent for IBD treatment.

## 1. Introduction

The chronic disease inflammatory bowel disease (IBD), consisting of ulcerative colitis (UC) and Crohn’s disease (CD), is complex because of relapsing disorders [1]. Once caught by IBD, patients will be beset by epithelial barrier disruption and mucosal ulceration, rectal bleeding, mucous stool, and diarrhea [2,3,4]. The pathogenesis of IBD is still unknown, however, the widely accepted hypothesis is that, in the development of IBD, a dominant factor is the immune response lead by an initial defect in sampling gut luminal antigens or a mucosal susceptibility [5]. Meanwhile, the impaired barrier functions of intestinal epithelial cells may also facilitate the development of inflammation due to the overt activation of immune response [6,7]. An immense number of studies showed that CD4+ T helper cells are crucial to the occurrence of IBD. Generally, CD, a Th1 cytokine-mediated disorder, is characterized by a high production of IFN-γ, TNF-α and IL-12, while UC, mediated by Th2 cytokines, is a disorder associated with increased production of IL-6 but decreased IL-10 production. These pro-inflammatory cytokines have a great importance in pathogenesis of IBD [8,9], and are used to be regarded as an indispensable indicator of curative effect of IBD [10,11]. Therefore, a large number of therapeutic agents are used to treat colitis via inhibiting inflammatory response, such as 5-aminosalicylates (5-ASA) and corticosteroids [12]. The recrudescence rates of IBD relatively high [12,13], however, a lot of drugs are limited in clinical application, and the irreversible and severe side effects of mainstream pharmaceuticals, should not be ignored. Therefore, it is urgent to find new potential molecules for IBD treatment, preferably of natural origin with high efficacy and safety.

Farrerol, a type of 2,3-dihydro-flavonoid, is extracted from Rhododendron. Studies have shown that Farrerol has been used to exert various pharmacological activities, such as anti-inflammatory [14], antibacterial [15], and antioxidant activity [16]. Furthermore, it has been reported that Farrerol provides protection against LPS-induced acute lung injury [17]. However, the effect of Farreol on colitis has not been reported. This study aimed to investigate the effects of Farrerol on TNBS-induced colitis in mice and to find a common potential mechanism of how it works in colitis.

## 2. Results

### 2.1. Farrerol Ameliorates TNBS-Induced Colonic Injury

**Figure 1:**
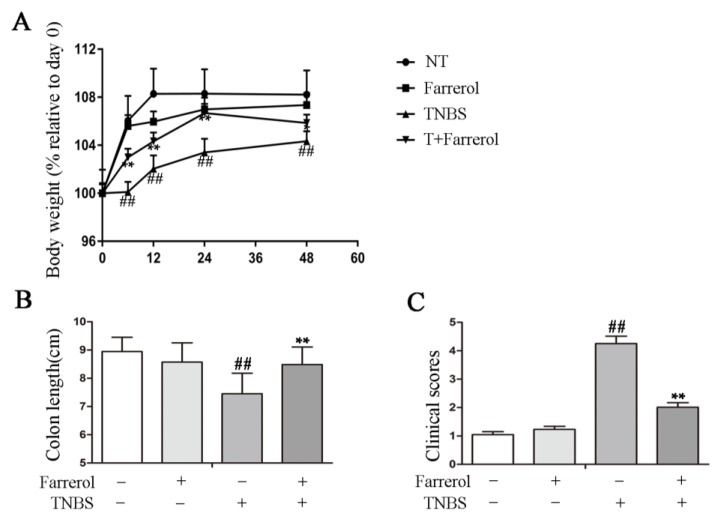
Farrerol ameliorates 2,4,6-trinitrobenzene sulfonic acid (TNBS)-induced colonic inflammation in mice. (**A**) Body weight. Body weights were normalized to body weight on day 0; (**B**) The colon length of mice with different treatment; and (**C**) Clinical score of mice. *n* = 10, and ^##^ *p* < 0.01 compared to the control group, ** *p* < 0.01 compared to the TNBS treatment group-statistical significance was determined by ANOVA.

To define the protective effects of Farrerol in TNBS-induced colitis, we measured the weight change, length of colon, and clinical score of the mice and observed the weight change of mice that was significantly increased following TNBS treatment for 6, 12, 24, and 48 h, respectively (Figure 1A). Farrerol administration had no effect on weight change when compared to no treatment mice but markedly decreased the weight change of TNBS-treated mice (Figure 1A). The protective effects were also observed in colon length and clinical score. TNBS-treated mice had a significantly decreased colon length and increased clinical score (Figure 1B,C). However, Farrerol treatment observably improved the colon length (Figure 1B) and the clinical score (Figure 1C). Collectively, these results indicated that Farrerol had a protective effect on TNBS-induced colon injury.

### 2.2. Farrerol Inhibits Inflammatory Response in TNBS-Induced Mice

**Figure 2:**
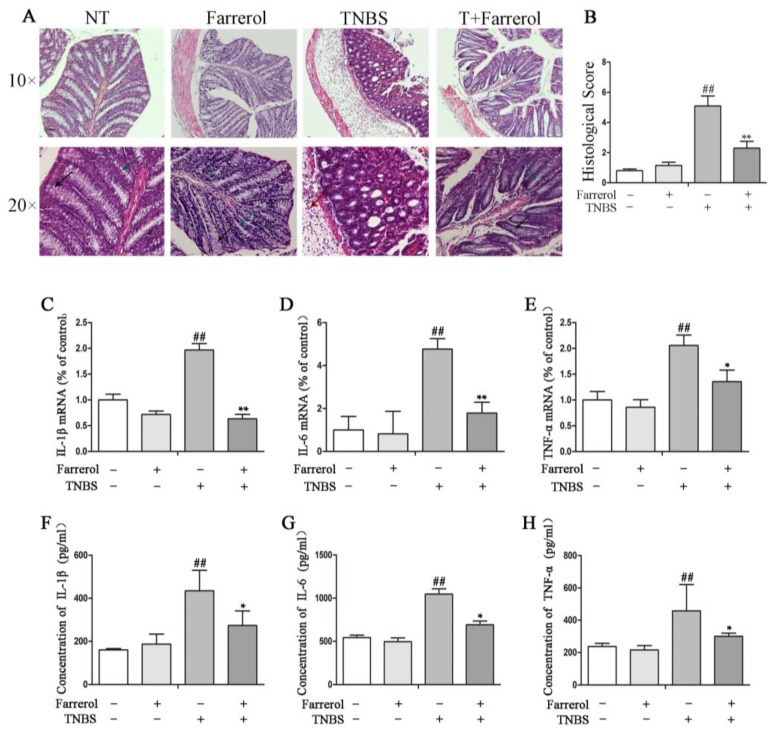
Farrerol inhibits inflammatory response in TNBS-induced mice. (**A**) hematoxylin and eosin (H&E) staining of colonic tissue from mice with different treatment. The **black** arrow represented goblet cells, **green** arrow represented crypts and **red** arrow represented infiltration of inflammatory cells. The scale bar represents 300 μm in 10×, and 100 μm in 20×; (**B**) Histological score of the colon. The gene expression of IL-1β (**B**), IL-6 (**C**), and TNF-α (**D**) in colonic tissue was examined by quantitative real-time PCR. The protein levels of IL-1β (**E**), IL-6 (**F**), and TNF-α (**G**) in colonic tissue homogenates were examined by ELISA. *n* = 10, ^##^ *p* < 0.01 compared to the control group, * *p* < 0.05 and ** *p* < 0.01 compared to the TNBS treatment group, statistical significance was determined by ANOVA.

To further explore the protective effects of Farrerol in TNBS-induced colitis in mice, we performed hematoxylin and eosin (H&E) staining and measured the expression of pro-inflammatory cytokines in colon from mice with different treatment. The results showed that TNBS-induced mice showed increased colonic damage characterized by a significant increased depletion of goblet cells and crypts, increased infiltration of inflammatory cells, severe submucosa edema, and disruption of colonic architecture when compared with the no treatment group and Farrerol administration group (Figure 2A). Therefore, histoligical score in TNBS-induced mice as well significantly increased (Figure 2B). Farrerol treatment did not alter the normal mucosal architecture but distinctly improved TNBS induced inflammatory activity, mucosal architecture disruption and histological score (Figure 2A,B). Additionally, TNBS-stimulated mice had a significant increased production of pro-inflammatory cytokines in colon tissue including IL-1β, IL-6, and TNF-α (Figure 2C–H). As expected, Farrerol markedly decreased the production of IL-1β (Figure 2C,F), IL-6 (Figure 2D,G), and TNF-α (Figure 2E,H) in TNBS-induced mice. These results indicated that Farrerol administration ameliorates TNBS-induced colitis.

### 2.3. Farrerol Maintains Mucin2 Expression and Appropriate Tight Junctions in the Colon of TNBS-Induced Mice

**Figure 3:**
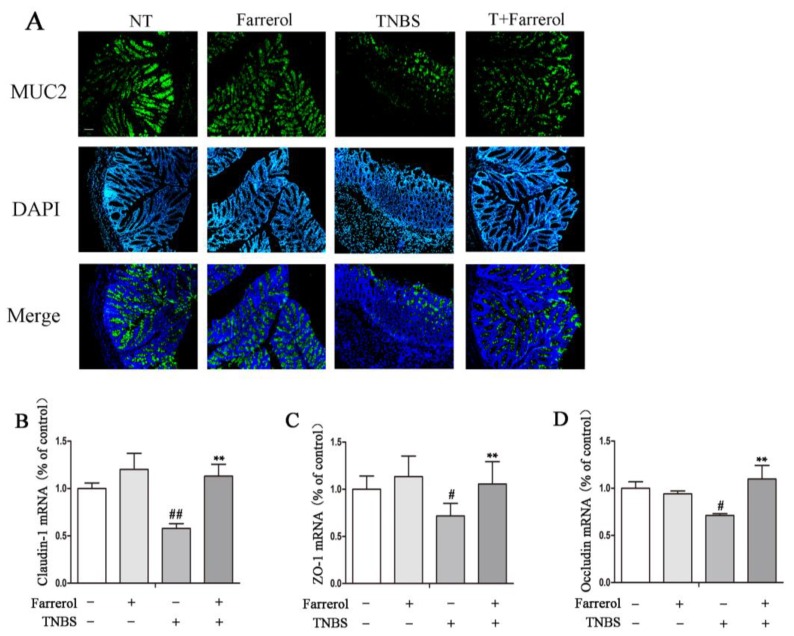
Farrerol maintains mucin-2 (MUC2) expression and proper tight junctions in TNBS-induced mice. (**A**) Colonic MUC2 immunofluorescence staining. The MUC2 was observed by epifluorescence microscope. Green represented MUC2, and Blue represented DAPI. The magnification is 10×, and the scale bar represents is 100 μm. The gene expression of tight junction proteins including claudin-1 (**B**), ZO-1 (**C**), and Occludin (**D**) in the colonic tissue. *n* = 10, ^#^ *p* < 0.05 and ^##^ *p* < 0.01 compared to the control group, ** *p* < 0.01 compared to the TNBS treatment group, and statistical significance was determined by ANOVA.

To further explore the protection effect of Farrerol in experimental colitis, we investigated the expression of mucin-2 (MUC2) protein by immunofluorescence. The results showed that Farrerol treatment maintained the proper MUC2 protein expression in TNBS-induced mice (Figure 3A). Quantitative real-time PCR was used to examine tight junction protein expression including claudin-1, ZO-1, and Occludin in colonic tissue at mRNA levels. The results showed that TNBS markedly reduced the expression of tight junction protein. However, Farrerol treatment significantly improved the expression of claudin-1 (Figure 3B), ZO-1 (Figure 3C) and Occludin (Figure 3D). Together, these results indicated that Farrerol had a beneficial effect on TNBS-induced mucosa disruption.

### 2.4. Farrerol Inhibits Inflammatory Response in LPS-Stimulated RAW264.7 Cells

**Figure 4:**
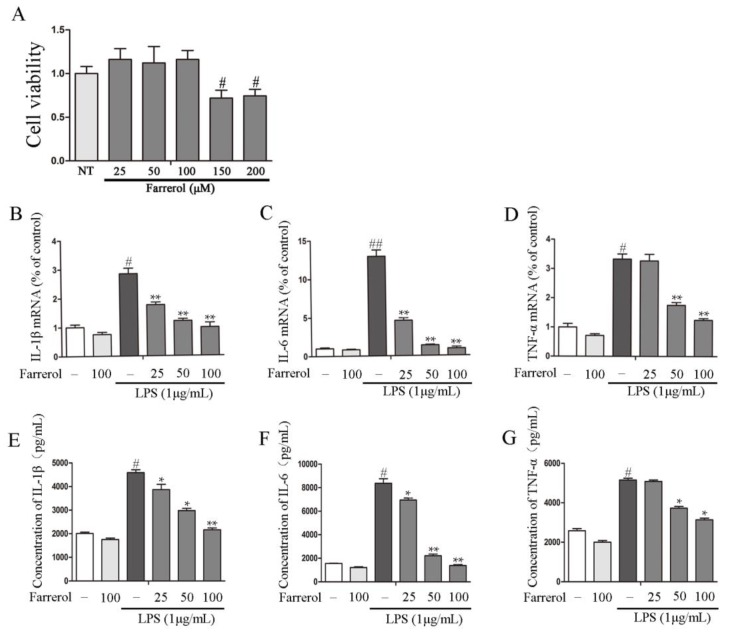
Farrerol inhibits inflammatory cytokine production in LPS-stimulated RAW264.7 cells. (**A**) The impact of Farrerol (25, 50, 100, 150, and 200 μM) on RAW264.7 cells viability. RAW264.7 cells were pretreated with different concentrations of Farrerol for 1 h, then stimulated with LPS (1 μg/mL) for 6 h. Quantitative real-time PCR was used to detect IL-1β, IL-6, and TNF-α production at mRNA levels (**B**–**D**) (*n* = 3). For ELISA analysis, the cells were treated with Farrerol for 1 h and then were stimulated by LPS (1 μg/mL) for 12 h (**E**–**G**) (*n* = 3). ^#^ *p* < 0.05 and ^##^ *p* < 0.01 compared to the control group, * *p* < 0.05 and ** *p* < 0.01 compared to the LPS group, and statistical significance was determined by ANOVA.

**Figure 5:**
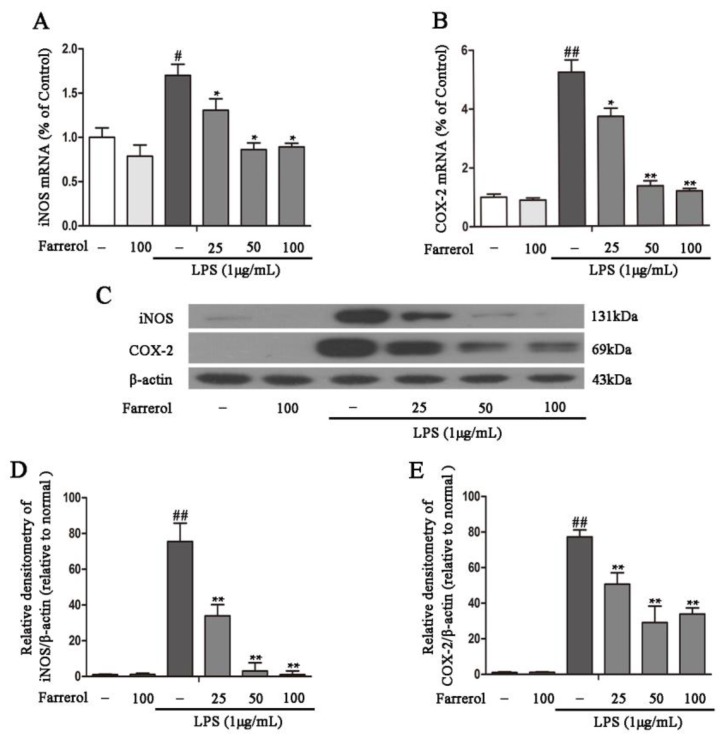
Farrerol inhibits inflammatory enzymes production in LPS-induced RAW264.7 cells. The cells were incubated with Farrerol for 1 h before LPS stimulated for 6 h or 12 h. Total RNA was extracted from cells by Trizol following LPS stimulated for 6 h. The mRNA levels of COX-2 and iNOS were examined by quantitative real-time PCR (**A**,**B**) (*n* = 3). The protein levels of COX-2 and iNOS were detected by western blot following LPS stimulated for 12 h (**C**–**E**) (*n* = 3). ^#^ *p* < 0.05 and ^##^ *p* < 0.01 compared to the control group, * *p* < 0.05 and ** *p* < 0.01 compared to the LPS group, and statistical significance was determined by ANOVA.

To further explore the potential mechanisms of Farrerol on colitis in vitro, LPS-stimulated RAW264.7 cells were used as an inflammatory cell model. First, we measured the effect of Farrerol on RAW264.7 cells viability, and the results showed that 25, 50, and 100 uM of Farrerol have no toxic effects on RAW264.7 cells (Figure 4A). Then, cells were treated with Farrerol for 1 h, followed by LPS stimulated for 6 h (for mRNA level) or 12 h (for protein level). The result indicated that Farrerol pretreatment has no effect on inflammatory cytokine production in non-treated cells but sharply decreases the inflammatory cytokines production including IL-1β (Figure 4B,E), IL-6 (Figure 4C,F), and TNF-α (Figure 4D,G) in LPS-treated cells in a concentration-dependent manner. To further determine that Farrerol inhibits inflammatory response, the expression of pro-inflammatory enzymes, COX-2 and iNOS, was measured by RT-PCR and Western blot. We found that LPS stimulation promoted the expression of COX-2 and iNOS. However, the pre-treatment with Farrerol significantly decreased the expression of COX-2 (Figure 5A,C,D) and iNOS (Figure 5B,C,E) in LPS-stimulated RAW264.7 cells.

### 2.5. Farrerol Inhibits the Activation of NF-κB and MAPK Signaling Pathways in LPS-Stimulated RAW264.7 Cells

**Figure 6:**
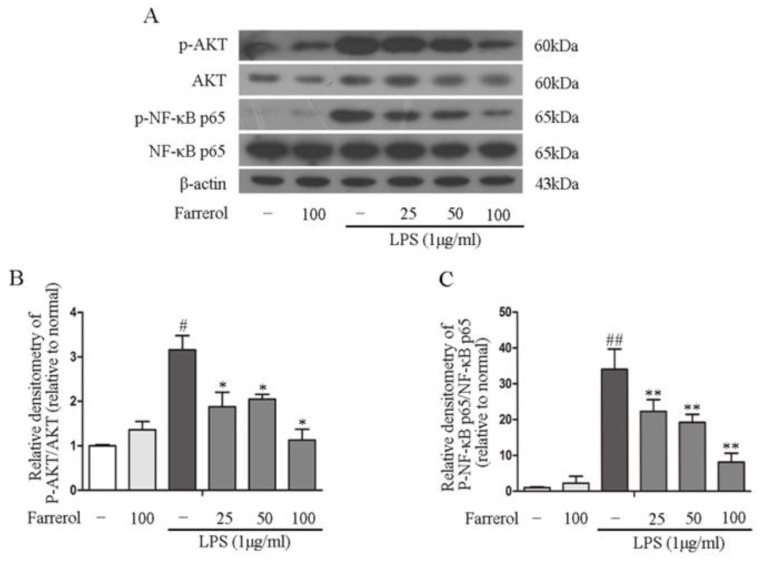
NF-κB p65 and AKT phosphorylation were downregulated by Farrerol in LPS-stimulated RAW264.7 cells. Pretreating cells with different concentrations of Farrerol (25, 50, and 100 μM) for 1 h, then cells were stimulated with LPS (1 μg/mL) for 1 h. The phosphorylation and total forms of NF-κB p65 and AKT were examined by western blot (**A**). The phosphorylation ratio of NF-κB p65 and AKT were quantified (**B**,**C**) (*n* = 3). ^#^ *p* < 0.05 and ^##^ *p* < 0.01 compared to the control group, * *p* < 0.05 and ** *p* < 0.01 compared to the LPS group, and statistical significance was determined by ANOVA.

**Figure 7:**
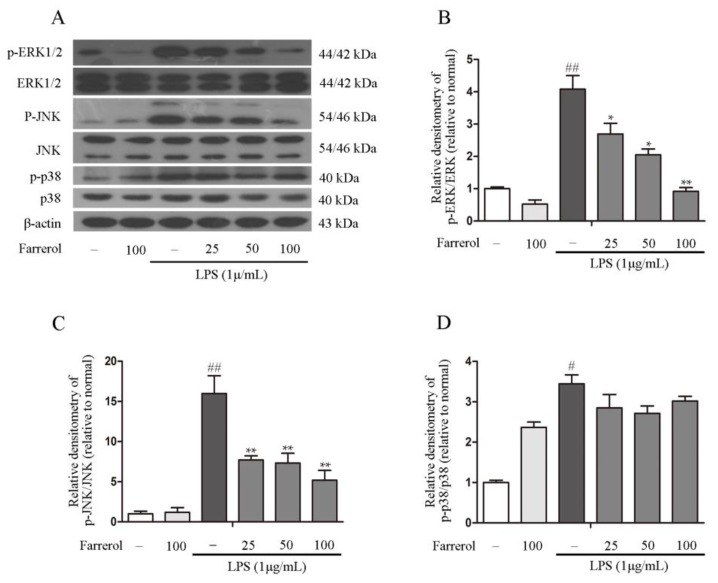
Farrerol inhibits ERK1/2 and JNK phosphorylation in LPS-stimulated RAW264.7 cells. Pretreating cells with different concentrations of Farrerol (25, 50, and 100 μM) for 1 h, then cells were stimulated with LPS (1 μg/mL) for 1 h. The phosphorylation and total forms of the MAPK signaling pathways were detected via western blot (**A**). The phosphorylation ratio of ERK1/2, JNK and p38 were quantified (**B**–**D**) (*n* = 3). ^#^ *p* < 0.05 and ^##^ *p* < 0.01 compared to the control group, * *p* < 0.05 and ** *p* < 0.01 compared to the LPS group, and statistical significance was determined by ANOVA.

To explore the potential mechanisms of the anti-inflammatory effect of Farrerol in TNBS-induced colitis, mitogen-activated protein kinases (MAPKs) and NF-κB phosphorylation are investigated in this experiment. The results showed that pretreating with Farrerol sharply decreased the levels of AKT and NF-κB p65 phosphorylation in LPS-stimulated RAW264.7 cells (Figure 6A–C). Furthermore, we observed that LPS stimulation can markedly increase p38, ERK1/2, and JNK phosphorylation; however, Farrerol markedly decreased the ERK1/2 and JNK1/2 phosphorylation but had no effect on the p38 phosphorylation in LPS-stimulated RAW264.7 cells (Figure 7A–D). Collectively, these results indicated that the anti-inflammatory effect of Farrerol in RAW264.7 was mediated by inhibiting AKT, NF-κB p65, ERK1/2, and JNK1/2 phosphorylation.

## 3. Discussion

IBD is mainly divided into the UC and CD [18], the representative symptoms of which are abdominal pain, fever, and weight loss. The symptoms of this disease are usually switching from light to severe in the course of illness [3,19]. Studies have reported that inflammation has a great importance in the development of IBD, especially UC. Current research on inflammatory treatment focuses on natural anti-inflammatory drugs because its side effects and virulence are relatively weak [20]. Evidences have shown that obtains from natural plants are highly effective in treating many inflammatory diseases such as colitis [21,22]. Farrerol, a new type of 2,3-dihydro-flavonoid, is obtained from Rhododendron. Previous studies have described that Farrerol attenuates the aortic lesion in hypertensive mice and has anti-bacterial and anti-inflammatory activities [23]. Farrerol has also been shown to provide protective effects in LPS-induced acute lung injury [17]. Therefore, we predict that the Farrerol might provide a protective effect in intestinal epithelium barrier integrity and might be a potential therapeutic molecular for IBD treatment.

The TNBS-induced IBD model has been widely used for studying colitis treatments that perfectly stimulates the development of colitis and exploring the therapeutic effects of potential agents [24]. TNBS is a covalently reactive compound that binds to autologous proteins and can stimulate delayed-type hypersensitivity to hapten-modified self-Ags, which involves and is regulated by complex interactions between various functional subsets of CD4+ T cells. These events significantly increased the disruption of the colonic structure, leading to the injury of intestinal barrier and CD4^+^ T cell-mediated immune response [25,26,27]. Weight loss, colon length, and elevated clinical score are the basic features of IBD [28,29,30]. In this research, we found that Farrerol significantly improved the weight change, length of colon, and reduced the clinical score in TNBS-induced mice. The H&E staining confirmed that Farrerol effectively ameliorated the reduction of goblet cells and crypts, inflammatory cells infiltration, and disruption of colonic architecture, which are crucial for the maintaining of normal colonic functions [31,32]. In the colon, goblet cells have an important function in mucin secretion, and overt inflammation can cause loss of goblet cells, which is one of the histopathology features of IBD. Overt intestinal inflammation also results in the disruption of epithelium such as depletion of crypt, consequently impairing the barrier function and leading to a disability in limiting bacterial translocation. In the inflamed intestines of CD and UC patients, several pro-inflammatory cytokines, such as TNF-α and IL-1β, have been found to be elevated. The suppression of cytokines production was the primary for the concept of treatment of IBD [33]. In this study, we found that supplemented with Farrerol significantly suppressed the production of pro-inflammatory cytokines TNF-α, IL-6 and IL-1β in TNBS-induced colitis mice.

In the subepithelial dome of Peyer’s patches, there are a large number of dendritic cells and macrophage that sense luminal contents. Over-activation of intestinal immunity is the main reason for stimulating inflammation and further aggravation in IBD. Study has shown that there is a significant increase in immune cell numbers that ultimately results in elevated inflammatory cytokines production [34]. CD is characterized by aggregation of large number of macrophages [35]. Macrophages have important regulatory effect in IBD development, which is frequently used to investigate mechanism of IBD in vitro [36,37]. To further determine the potential anti-inflammatory effects of Farrerol in IBD, we detected the impact of Farrerol on inflammatory responses in LPS-stimulated RAW264.7 cells. As we have expected, Farrerol significantly decreased the production of pro-inflammatory mediator IL-1β, IL-6, TNF-α, COX-2, and iNOS [38,39]. All the results indicated that Farrerol provide anti-inflammatory effects in TNBS-induced IBD.

The inflammatory response caused by the up-regulation of inflammatory mediators is a main factor of intestinal barrier disruption and dysfunction [40]. Therefore, we further examined the effects of Farrerol on intestinal epithelial barrier function in TNBS-induced mice. Tight junctions maintain the integrity of intestinal epithelial barrier. The increase of intestinal permeability and the dysfunction of intestinal epithelial barrier play a key role in the pathogenesis of IBD [4]. In this study, we found that Farrerol significantly improved the expression of claudin-1, occludin, and ZO-1 in colonic tissue from TNBS-induced mice. The mucus layer is mostly composed of mucin, defensin, and lecithin that is abundant in the colon and covers the outer epithelial surface to protect the mucosa [41]. The mucus layer contains an outer loose permeable layer that contacts commensal bacterial and an inner firm mucus layer that contacts the intestinal epithelium cells [42]. The reduction of mucin secretion would leave the intestinal mucosa exposed to the intestinal pathogens and increase the incidence of IBD. The results of immunofluorescence staining showed that Farrerol maintains proper mucin-2-expression in TNBS-induced mice. From these results, we can conclude that Farrerol contributes to the maintenance of the intestinal epithelial barrier integrity in TNBS-induced mice.

NF-κB p65 has been found to participate in inflammatory cytokines production [43]. AKT, the upstream molecules of NF-κB, also plays a vital role in NF-κB activation [44,45]. To survey the potential mechanism of Farrerol in inflammatory responses, we monitored the effect of Farrerol on the activation of AKT and NF-κB p65 and observed that Farrerol significantly reduced LPS-induced AKT and NF-κB p65 phosphorylation in RAW246 cells. MAPK pathways is also involved in regulation of inflammatory cytokines production [46,47]. Therefore, we further supervised the effects of Farrerol on p38, ERK1/2, and JNK1/2 phosphorylation in LPS-induced RAW264.7 cells. What came out is that Farrerol significantly inhibited ERK1/2 and JNK1/2 phosphorylation but has no effect on p38 activation. Previous study showed that Farrerol significantly inhibited the phosphorylation of p38 signaling pathway in LPS-induced mastitis. However, Ci et al. indicated that Farrerol has no effects on the phosphorylation of p38 in an OVA-induced allergic asthma and LPS-induced acute lung injury [17]. Therefore, we thought that Farrerol might play a different function on the p38 signaling activation in different cells which need to be further investigated.

In conclusion, our results demonstrated that Farrerol improve the weight loss, colon length and clinical score in TNBS-induced colitis mice. Furthermore, we found that Farrerol inhibited the expression of pro-inflammatory mediators and contributed to the maintenance of the intestinal epithelium barrier integrity in TNBS-induced colitis mice. These anti-inflammation effects may be related to the inhibition of Farrerol on the phosphorylation of AKT, NF-κB, ERK1/2 and JNK1/2. This study indicated that Farrerol may be a potential therapeutic agent in the treatment of IBD.

## 4. Materials and Methods

### 4.1. Animals and Colitis Induction

Six- to eight-week-old C57BL/6 mice were bred in the center of experimental animal and subordinated to Bethune Medical College of Jilin University (Changchun, China) (approved on 27 February 2015, Permit Number: 2015047). The mice were cultivated in a house at 23–24 °C and 12 h day and 12 h night. All the mice were divided into four groups: no treatment group, Farrerol treatment group, TNBS-induced colitis group, and TNBS-induced mice pretreated with Farrerol group. All the mice of each group were cultured in the same cage and fasted for 24 h prior to the induction of colitis. Then, mice were anesthetized with ether. Two mg of TNBS in the 100 μL of 50% ethanol solution was administered into the colon through a catheter inserted approximately 3 cm into the anus. The Farrerol (45 mg/kg/day) was orally administrated by an 8G lavage needle (Times, Shanghai, China) from three days before TNBS infusion to the day before mice were killed. The body weight was recorded at 0, 6, 12, 24, and 48 h after TNBS treatment. Then, mice were sacrificed by cervical dislocation to evaluate the colon length, clinical score, intestinal inflammation, and intestinal epithelial barrier.

### 4.2. Clinical Score

During the experiment, roachback or emaciation, colon thickness, and pellet morphology were recorded. Clinical scoring system is shown in Table 1.

### 4.3. Hematoxylin and Eosin (H&E) Staining

In this experiment, the middle colon was saturated into 4% buffered formalin solution, dehydrated with grade ethanol, and paraffin was used to embed the colon. Then, the colon was cut into 5 μm-thick sections and stained by H&E [48]. All sections after H&E staining were assessed of histological change by optical microscope. Infiltration of inflammatory cells, disappearance of goblet cells, and disruption of colonic architecture were criteria for assessing histological score as previously described [49].

### 4.4. Quantitative Real-Time PCR

Total RNA was extracted from mice colonic segments and RAW264.7 cells using Trizol (Invitrogen, Carlsbad, CA, USA). A commercial RT-PCR Kit (Takara Shuzo Co., Ltd., Kyoto, Japan) was used for mRNA (2 mg) reverse transcription in a final volume of 20 μL. RT-PCR was performed using a SYBR Green PCR Master Mix (Roche, South San Francisco, CA, USA). The primer sequences are shown in Table 2.

### 4.5. ELISA

RAW264.7 cells were pretreated with Farrerol for 1 h and then stimulated with LPS (1 μg/mL). The culture medium was collected 12 h later. Colon tissues were homogenized in PBS (4 mL/g tissue) and centrifuged at 4 °C and the supernatant was collected. The concentration of IL-1β, IL-6, and TNF-α in culture medium and supernatant were measure following the instructions of the manufacture (Biolegend, San Diego, CA, USA).

### 4.6. Immunofluorescence

Five-μm sections staining. First, the paraffin was removed by using diethyl ether, and then dehydration was performed with grade ethanol. The sections were boiling in sodium citrate buffer to expose the antigens, and the sections were cooled at room temperature and washed with PBS three times for more than 5 min. The sections were coated by donkey serum at room temperature for 1 h and washed with PBS three times. Then, the sections were incubated with primary antibody (Mucin-2 H-300, 1:200, Santa Cruz, CA, USA) at 4 °C overnight. The next day, the sections were washed with PBS three times and incubated with the secondary goat anti-rabbit antibody (1:2000; Santa Cruz, CA, USA) at room temperature for 1 h and then subsequently washed with PBS three times and followed by Nuclei staining with DAPI.

### 4.7. Cell Culture and Treatment

RAW264.7 cells were acquired from BeNa Culture Collection (Beijing, China) and brooded in DMEM with 10% FBS. RAW264.7 cells were seeded into 6-well (1.0 × 10^6^/well) or 24-well (0.5 × 10^6^/well) plates overnight and pretreated with Farrerol for 1 h followed by LPS (1 μg/mL) treatment for indicated time points. Subsequently, RAW264.7 cells were collected and used to measure the reflections of pro-inflammatory mediators and the phosphorylation of signaling pathway by quantitative real-time PCR- and ELISA-analysis, respectively.

### 4.8. MTT

Cell viability was measured by MTT. In this study, 5 × 10^3^ RAW264.7 cells were seeded into 96-well plates in a volume 200 μL/well. After 24 h in 37 °C and 5% CO_2_, 200 μL of DMEM with 10% FBS was replaced to each well for 1 h, and then cells were treated with different concentrations of Farrerol (25 μM to 200 μM) and incubated for 24 h. To each well was added a mixture of 100 μL (the ratio of MTT to DMEM is 1/9) for 4 h. The liquid of all wells was removed and added to 150 μL DMSO for 15 min in an oscillator. Absorbance was measured at 570 nm on a microplate reader.

### 4.9. Western Blot

For this stage, 1.5 × 10^6^ of RAW264.7 cells were seeded in the 6 cm culture dish. Two days later, the cells were pretreated with Farrerol for 1 h and then stimulated with LPS for 1 h. RAW264.7 cells were lysed by RIPA buffer (Roche Diagnostics, Swissland) on ice for 15 min. A bicinchoninic acid protein assay kit (Beyotime Inst. Biotech, Shanghai, China) was used to measure protein concentration in the samples. Subsequently, 50 μg proteins were separated through 10% or 12% SDS-PAGE and then transferred to PVDF membranes. After blocking in 5% non-fat milk dissolved in Tris buffered saline for 1 h, the membranes were incubated in primary antibodies against iNOS (1:4000), COX-2 (1:2000), phosphor-ERK1/2 (1:1000), ERK1/2 (1:1000), phosphor-p38 (1:1000), p38 (1:2000), phosphor-JNK1/2 (1:2000), JNK 1/2 (1:2000), phosphor-NF-κB p65 (1:2000), NF-κB p65 (1:2000) (Cell Signaling Technology, Danvers, MA, USA), and β-actin (1:2000) (Santa Cruz Biotechnology Inc., Santa Cruz, CA, USA) at 4 °C overnight. Subsequently, the membranes were washed for three times and incubated with the secondary goat anti-rabbit antibody (1:2000; Santa Cruz, CA, USA) for 1 h. By enhancing chemiluminescence, membranes could be visualized. (ECL kit; Applygen Inst. Biotech, Beijing, China).

### 4.10. Statistical Analysis

Results are shown as mean ± SD of at least three replicates. The statistical software package of SPSS 12.0 (SPSS Inc., Chicago, IL, USA) improves the quality of the date analysis. One-way analysis of variance (ANOVA) was performed on the group, followed by the minimum difference test. A value of *p* < 0.05 is considered to be statistically significant.

## Figures and Tables

**Table 1:** The scoring system of clinical score.

Score	Roachback or Emaciation	Colon Thickening	Pellet Morphology
0	None	None	Normal
1	Yes	Slight	Soft stool
2		Moderate	Diarrhea
3		Severe	Bloody stool

**Table 2:** Primers used for quantitative real-time PCR.

Gene	Sequence	Length (bp)
***β-actin***	F: 5′-GTCAGGTCATCACTATCGGCAAT-3′	147
	R: 5′-AGAGGTCTTTACGGATGTCAACGT-3′	
***TNF-α***	F: 5′-CCACGCTCTTCTGTCTACTG-3′	136
	R: 5′-CCACGCTCTTCTGTCTACTG-3′	
***IL-1β***	F: 5′-TGTGATGTTCCCATTAGAC-3′	139
	R: 5′-AATACCACTTGTTGGCTTA-3′	
***IL-6***	F: 5′-AGCCACTGCCTTCCCTAC-3′	138
	R: 5′-TTGCCATTGCACAACTCTT-3′	
***iNOS***	F: 5′-CACCCAGAAGAGTTACAGC-3′	158
	R: 5′-GGAGGGAAGGGAGAATAG-3′	
***COX-2***	F: 5′-GGAGGGAAGGGAGAATAG-3′	127
	R: 5′-CTTGTAGTAGGCTTAAACATAG-3′	
***Claudin-1***	F: 5′-AGGTCTGGCGACATTAGTGG-3′	204
	R: 5′-CGTGGTGTTGGGTAAGAGGT-3′	
***Occludin***	F: 5′-GACCTTGATTTGCATGACGA-3′	197
	R: 5′-AGGACCGTGTAATGGCAGAC-3′	
***ZO-1***	F: 5′-ACACTTGCTTGGGACAGAGG-3′	199
	R: 5′-AAGGAAGCGATGAAGCAGAA-3′	

## Abbreviations

IBD	Inflammatory bowel disease
CD	Crohn’s disease
UC	Ulcerative colitis
TNBS	2,4,6-Trinitrobenzene sulfonic acid
LPS	Lipopolysaccharide
